# On Response Bias in the Face Congruency Effect for Internal and External Features

**DOI:** 10.3389/fnhum.2017.00494

**Published:** 2017-10-17

**Authors:** Günter Meinhardt, Bozana Meinhardt-Injac, Malte Persike

**Affiliations:** Department of Psychology, Johannes Gutenberg University Mainz, Mainz, Germany

**Keywords:** feature integration, congruency effect, response bias, selective attention

## Abstract

Some years ago Cheung et al. (2008) proposed the complete design (CD) for measuring the failure of selective attention in composite objects. Since the CD is a fully balanced design, analysis of response bias may reveal potential effects of the experimental manipulation, the stimulus material, and/or attributes of the observers. Here we used the CD to prove whether external features modulate perception of internal features with the context congruency paradigm (Nachson et al., 1995; Meinhardt-Injac et al., 2010) in a larger sample of *N* = 303 subjects. We found a large congruency effect (Cohen's *d* = 1.78), which was attenuated by face inversion (*d* = 1.32). The congruency relation also strongly modulated response bias. In incongruent trials the proportion of “different” responses was much larger than in congruent trials (*d* = 0.79), which was again attenuated by face inversion (*d* = 0.43). Because in incongruent trials the wholes formed by integrating external and internal features are always different, while in congruent trials same and different wholes occur with the same frequency, a congruency related bias effect is expected from holistic integration. Our results suggest two behavioral markers of holistic processing in the context congruency paradigm: a performance advantage in congruent compared to incongruent trials, and a tendency toward more “different” responses in incongruent, compared to congruent trials. Since the results for both markers differed only quantitatively in upright and inverted presentation, our findings indicate no change of the face processing mode by picture plane rotation. A potential transfer to the composite face paradigm is discussed.

## 1. Introduction

When humans have reached high levels of expertise with individual members of an object category they have difficulty to judge object parts independently (Gauthier and Tarr, 2002; Chua et al., 2014). Particularly, this is true for human faces (Gauthier and Tarr, 1997; Gauthier et al., 2003). The strong contextual influence may reflect “holistic” processing—the tendency to process faces as indecomposable wholes (Rossion, 2008, 2013). Joint processing of face parts makes face processing highly efficient in various tasks, such as recognition (Richler et al., 2010; Wang et al., 2012; DeGutis et al., 2013), discrimination (Ellis et al., 1979; Richler et al., 2009; Meinhardt-Injac, 2013), and visual search (Hershler and Hochstein, 2005). However, it is disadvantageous when individual facial details have to be judged, since perception of these details contingently changes with the embedding facial context. The failure of selective attention to parts thus offers methodological access to the principles of feature integration in face perception (see Maurer et al., 2002; Richler and Gauthier, 2014 for overviews).

**Figure 1:**
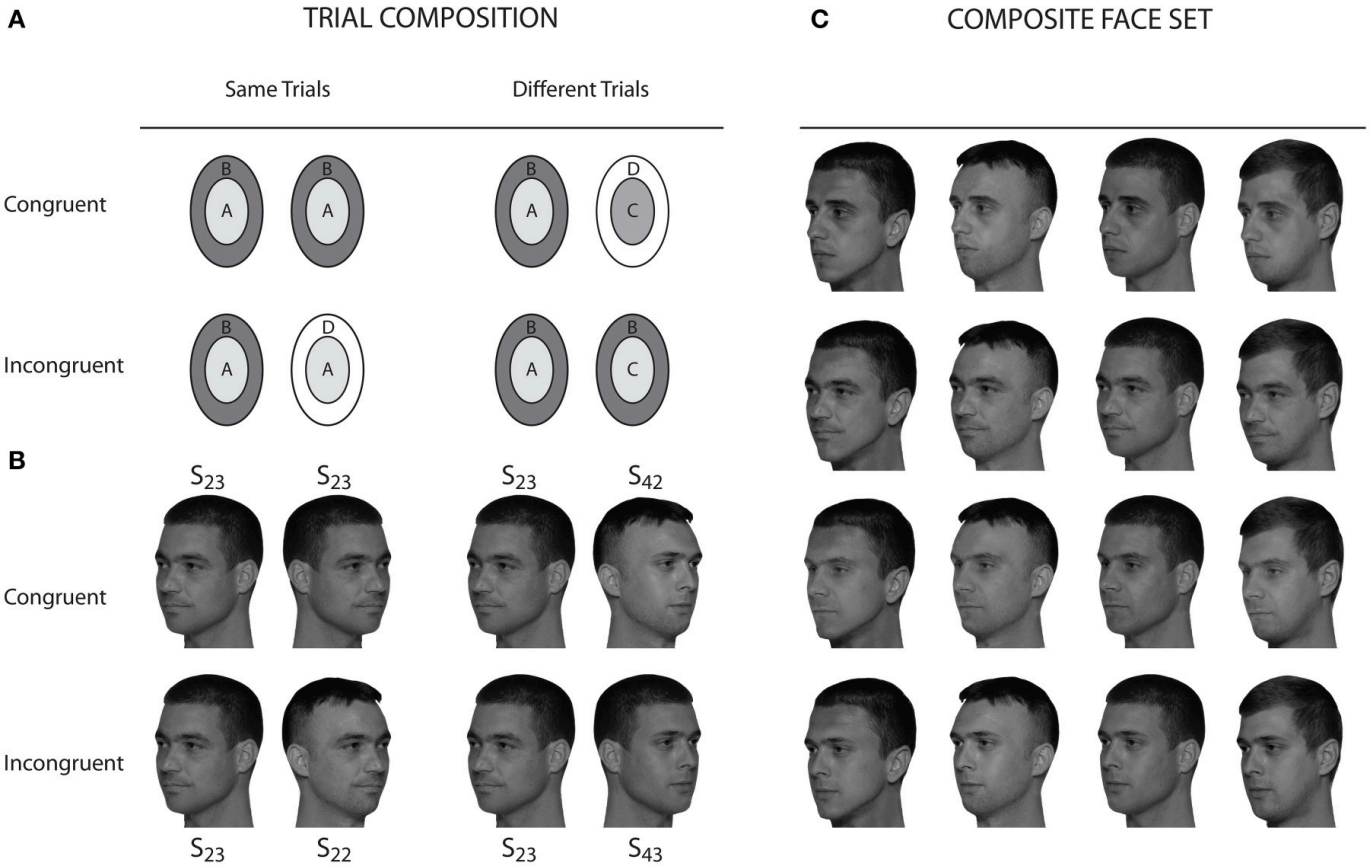
The context congruency paradigm in the framework of the complete design. In **(A)** the construction principle for same and different trials is illustrated. The context congruency paradigm employs internal and external features. The example shown here illustrates the variation of the congruency relation for internal feature matching (“attend inner”). In congruent trials, both the attended and the unattended parts are same in “same” trials and different in “different” trials (total sameness and difference). In incongruent trials, the unattended parts are different when the attended ones are same (“same” trial), and vice versa (“different” trial). **(B)** shows an example for a complete set of trials with a selection of stimulus instances from the composite face set, which is shown in **(C)**. In this set, the internal facial features are same in a line, while the external features are same in a column. In the notation *S*_*ij*_ the first index refers to line (internal features), and the second to column (external features). The left diagonal contains the original faces where all composites where constructed from. In the context congruency paradigm swapped 3/4 views are used for the first and second image presentation. All photo models agreed to scientific use and publication of their pictures.

In the last decade Cheung et al. (2008) proposed the complete design (CD) for measuring the failure of selective attention in same/diffferent discrimination tasks with composite objects. Originally, the CD was proposed for using it with the composite face paradigm, in which composite faces are created by combining upper and lower halves, and one class of halves has to be attended, while the other one has to be ignored. However, the CD can be applied to any kind of composite objects which fall into two complementary sets of target and non-target features. Accordingly, Meinhardt-Injac et al. (2010) used the CD for faces composed of complementary sets of external and internal features [“context congruency paradigm,” first introduced by (Nachson et al., 1995), but without using the CD], and also for other dichotomous compositions of face stimuli (Meinhardt-Injac et al., 2011). Figure 1 illustrates the CD and its trial types for the context congruency paradigm.

The crucial manipulation in the CD is the variation of the *congruency relation* among attended and non-attended parts. In congruent trials, there is complete identity or nonidentity of both composite objects: attended and non-attended parts are either both same (“same” trial) or both different (“different trial”). In incongruent trials, there is part-based identity/nonidentity. When the two objects agree in the attended parts, they disagree in the unattended parts (“same” trial), and vice versa (“different” trial). Therefore, in congruent trials, responding to the non-attended parts or to the attended parts is equivalent with respect to decision. In incongruent trials, however, the non-attended parts vary orthogonal to the attended parts, thus responding to the non-attended parts or to the attended parts results in opponent decisions. To measure performance a sensitivity measure is calculated from both response categories (usually *d*′), and the performance difference for congruent compared to incongruent trials (“congruency effect,” CE) is taken as an index for the failure of selective attention to parts (Richler et al., 2008).

The most advantageous feature of the CD is that it is fully balanced. The relative frequency of same and different pairs is the same for attended and non-attended parts. This means that a potential preference of the observer toward one response category (i.e., “response bias”) does not root in the formal characteristics of the design. This implies that response bias can be linked to the experimental manipulation, the stimulus material, and/or attributes of the observers. Particularly, a congruency effect on response bias is expected from holistic integration. We call this effect “congruency bias,” or CB, in close analogy to the congruency effect in the sensitivity measure, CE. *Both* congruency related effects, the CE *and* the CB, are implied by holistic integration when the CD is used.

Note that, in congruent trials, the wholes formed by integrating attended and non-attended parts are same in “same” trials and different in “different” trials. Since both occur with equal frequency, no bias is expected in congruent trials when the observer responds to perceived sameness or difference of the whole stimuli. In incongruent trials, however, the wholes formed by integrating attended and non-attended parts are *always* different, both in “same” trials and in “different” trials. Hence, if the integrated wholes rather than the parts drive the comparison of the two face stimuli, the observer should exhibit a strong tendency toward “different” responses. This means that we expect more “different” responses in incongruent compared to congruent trials when the observer processes face stimuli holistically. Thus, the CD ensures that the CB is a crucial test of holistic integration: a CB follows from holistic integration, while a failure to find a CB would indicate that *not* the integrated wholes drive the observers' responses. If, on the other hand, attended and non-attended parts were processed independently, then aberrations of the attentional focus toward the non-attended parts should occur when these agree or disagree, and with equal likelihood in either case. This means that the proportion of wrong “same” and wrong “different” responses in incongruent trials should be equal, i.e., there should be no CB. On the other hand, at least a moderate CE is expected from independent part processing, since a loss of attentional focus, which may occasionally occur even if the observers have good capabilities of attentional control, leads to errors in incongruent but not in congruent trials. Such errors contribute to a CE, but indicate a failure of selective attention which is not due to holistic processing. This means that the CB is apt to characterize the failure of selective attention qualitatively: If we observe a CE along with a CB in the complete design, then this strongly indicates that the observer is biased by properties of the wholes rather than of single parts when she/ he judges face identities. A CE without a CB, on the other hand, would indicate that the properties of the wholes do not interfere with decision stronger than any other roots of reduced attentional control. Hence, a CE without a CB, even if the CE is a large effect, would not warrant concluding that holistic integration is the prevalent face processing mode. However, both effects, the CE and the CB, vary contingently with experimental conditions, and particularly, with properties of the face set. We turn to potential reasons for both error types in the section 4.

The question arises whether holistic integration implies a “different” bias in incongruent, but not in congruent trials in *absolute terms*, i.e., that the frequency ratio of “different” responses to “different” and “same” responses is larger than 0.5. The answer is *no*, since there may be more sources of influence on response bias in a same/different forced choice comparison of stimuli. Properties of the face set used for testing can drive a response bias, and also properties of the tested sample of observers. For example, it was found recently that older adults showed a strong overall “same” bias, which was stronger for non-face objects than for faces (Meinhardt et al., 2016). These effects may exist, and may add to the effects of manipulating the congruency relation. Even if there is a general “same” or “different” bias, there should be more “different” responses in incongruent, compared to congruent trials for stimuli which are processed holistically^1^.

Above we stressed the particular importance of using a fully balanced design like the CD. Indeed, only if there is no confound of trial type (“same” or “different”) with formal characteristics of the design the CB is diagnostic of holistic integration. Cheung et al. (2008) have pointed out that in many applications of the composite face paradigm the so-called “partial” design (PD) was used. The partial design is a subset of the CD where “different” trials are realized only in the congruent variety while “same” trials are always incongruent. Hence, in the PD 75% of all face half pairings are different and 25% are same. This confound may introduce an artificial bias toward “different” responses, which implies that more “different” than “same” responses are no conclusive evidence for holistic integration when the PD was used. A preference of “different” over “same” responses may merely reflect a likely decisional strategy in the PD: the observer adjusts the response criterion to perceived target/non-target likelihoods, which are biased by the frequency distribution of the non-attended parts. In Appendix B of Supplementary Material it is shown that a “different” bias results as a consequence of the attempt of the observer to maximize the proportion of correct judgements if she/he estimates that there is a larger a-priory probability of “different” events. However, if response bias is to be analyzed as dependent variable, the design itself must not contain confounds that may affect its magnitude^2^.

In this study we used the CD to substantiate holistic integration of external and internal facial features based on both the CE and the CB. Several studies have shown that face recognition, assessed by the identity of the internal features, is modulated by exchanging the external features (Sinha and Poggio, 1996; Andrews and Thompson, 2010; Andrews et al., 2010; Axelrod and Yovel, 2010), which indicates that there is integration of internal and external features in the observer's face stimulus representation. As argued above, this should translate not only into a CE, but also into a CB effect when the complete design is used. The CB, however can be expected to be smaller than the CE, since more errors of any kind in incongruent, compared to congruent trials make the CE, while only more errors of just one kind (i.e., wrong “different” responses) make the CB. Therefore, we decided to use a comparably large sample of *N* = 319 subjects, which enabled us to localize CE and CB measures, and their effect sizes, with good statistical precision.

Since the CB measures the particular tendency of the observer to respond to perceived sameness and difference of the integrated wholes, a potential extinction of this tendency by picture plane rotation of the face stimuli would indicate a change in the information the observer relies on when she or he compares faces. In one view of the face inversion effect it has been claimed that face inversion changes the reliance of the observer from configural-relational information toward feature-based, or part-based information (Diamond and Carey, 1986; Rhodes, 1988; Leder and Bruce, 2000; Rossion and Gauthier, 2002), which implies that inversion changes the routes involved in face processing (Rossion, 2008; Rossion and Boremanse, 2008). However, this issue is highly controversial. Riesenhuber and colleagues (Riesenhuber and Wolff, 2009) criticized the conceptual weakness of “featural” and “configural” image manipulations, and showed that face processing in both orientations can be explained by assuming the same pathways of complex image analysis (Riesenhuber et al., 2004). Studying the composite effect with the CD showed that a significant congruency × alignment interaction was preserved with inverted faces, though attenuated compared to upright presentation (Richler et al., 2011). Here, we add testing whether both measures of holistic processing, the CE and the CB, survive inversion of face images. To anticipate the findings, we obtained results that closely resembled the findings of Richler et al. (2011). Albeit smaller than for upright presentation, there were still CE and CB effects for inverted faces, giving rise to concluding that face inversion may reduce but does not abolish feature integration in face processing.

## 2. Materials and methods

### 2.1. Study outline

The experimental face perception test with the context congruency paradigm was part of a larger testing battery for testing perceptual, social cognition, and general cognitive abilities. The battery comprised 16 brief experimental tests and 4 questionnaire assessments. Testing was executed in two 90 min sessions at 2 consecutive days. The context congruency test was conducted at the first day of testing, and was among other tests of face perception and face recognition. The order of the tests was counterbalanced. Testing with each test of the battery lasted about 10–15 min.

### 2.2. Participants

Three hundred and nineteen subjects participated in the study. All participants were undergraduate students of the Johannes Gutenberg University Mainz. The age range was 17–37 years, mean age = 22.8 years, standard deviation = 3.4 years. 70.8% were female. The subjects had normal or corrected to normal vision, using corrective lenses in the latter case. All subjects received a global information about the elements of social cognition and the domains being assessed with the test battery. They were given a fee of 30 euros for participation at the two testing days.

### 2.3. Apparatus

The experiment was executed with Inquisit runtime units. Stimuli were displayed on NEC Spectra View 2040 TFT displays in 1, 280 × 1, 024 resolution at a refresh rate of 60 Hz. Screen mean luminance *L*_0_ was 100 cd/m^2^ at a michelson contrast of (*L*_*max*_ − *L*_*min*_)/(*L*_*max*_ + *L*_*min*_) = 0.98. No gamma correction was used. The room was darkened so that the ambient illumination matched that of the screen. Stimuli were viewed binocularly at a distance of 70 cm. Subjects used a distance marker but no chin rest throughout the experiment. Subjects provided responses on an external key-pad. No feedback about correctness was provided.

### 2.4. Stimuli

Photographs of four male face models were used as templates for stimulus construction. These were full-color 3/4 view photographs of the left face side captured in a photo studio under controlled lighting conditions, and using the same background for all photographs. Photographs were converted to 8 bit grayscale pictures. We employed 3/4 view photographs in left and right side perspective, the latter obtained by mirroring the original left face side photographs. Swapped 3/4 views were used to preclude using pictorial matching strategies, since the 3/4 view was shown to be the only view which ensures good generalization across views in face identity matching tasks (Hill et al., 1997). None of the models was wearing glasses, jewelry, or had a beard. Haircut and overall type appearance was chosen to be similar. All face models had comparable overall head geometry. The original images were manipulated with Adobe Photoshop in order to construct sample stimuli with defined combinations of internal and external features, with the objective to form natural looking composite face stimuli ensuring that original faces and composite faces where indistinguishable. Because models had similar head geometry it was possible to construct a cutting template for the internal features that enabled us to interchange the internal features among the four face models without changing forehead height or chin length. Mean gray level and contrast of each original internal feature template were measured, and were used as reference values for replacement. As a scheme for stimulus construction, we used a 4 × 4 composite face matrix with line index referring to internal features and column index referring to external features, such that an entry *S*_*ij*_ denotes a face with internal features of face *i* and external features of face *j*. Before placing the internal features of face *j* onto face *i*, mean gray level and contrast of the internal features of face *j* were adjusted to the values of the internal features of face *i*. As a result, internal features were smoothly integrated into the external feature surround. The composite face matrix is shown in Figure 1C. This matrix allowed us to form 16 identical face stimulus pairs (to be used for same-congruent trials, see Figure 1). For each of these 16 pairs it is possible to combine in nine different ways to form a complete sets of trials within the CD. Hence, with 16 replications of trial types, it was warranted that there was no repetition of the same stimulus pair, and that each subject responded to an individual choice of trial selections. Stimulus size was 300 × 400 pixels (width × height), which corresponded to 12 × 15 cm, or 9.65° × 12° visual angle. For each stimulus an individual mask was constructed from randomly ordered 5 × 5 pixel blocks of the stimulus image. Masks subtended 450 × 600 pixels (width × height). For more details and pictures of stimuli see Meinhardt-Injac (2013).

### 2.5. Procedure

A same/different forced choice matching task was used. The dependency of performance on exposure duration was known from former experiments on the effects of exposure duration using the same paradigm and the same stimuli (Meinhardt-Injac et al., 2010, 2011; Meinhardt-Injac, 2013). We selected an exposure duration of 433 ms, because performance proved to have certainly saturated for display times of beyond 400 ms. This assured that results were not confounded with potential speed differences among the observers, and that the reported effects were not transient effects, which may arise for brief presentation times, but vanish afterwards^3^. The subjects were informed that two faces images would be successively presented, one in left-hand sided and one in right-hand sided 3/4 view, that presentation could be upright or inverted, and that the identity of just the inner face parts had to be judged. They were also told that the external face parts could vary or be identical. The structure of a trial was: fixation mark (300 ms)—blank (100 ms)—first face stimulus (433 ms)—mask (350 ms)—blank (200 ms)—second face stimulus (433 ms)—mask (350 ms)—blank frame until response. The center positions of each of the two face images were shifted by 20 pixels away from the center in random direction to preclude that the same image parts were focused. The subjects were made familiar with the task by 16 randomly selected probe trials. Congruent and incongruent face half pairings were measured with 32 trials each, 16 were “same” and 16 were “different” trials. In half of the trials the first face image was in left-hand sided view, and in the other half in right-hand sided view. Further, in half of the trials all face images were presented upright, and inverted in the other half. The 128 trials were ordered randomly.

### 2.6. Dependent measures and data transformations

For the same/different experiment the “same” response category was defined as the target category. Accordingly, a correct “same” response was defined to be a hit, a wrong one a false alarm (error type I), a correct “different” response was denoted a correct rejection and an incorrect “different” response a miss (error type II). We abbreviate the rates for these events, i.e., *Hit, FA, CR, Miss*. The sensitivity measure *d*′ was calculated according to

(1) $$\begin{array}{l} d^{\prime }=z\left(Hit\right)-z\left(FA\right). \end{array}$$

(see MacMillan and Creelman, 2005, p. 8). In Equation (1), *z* is the quantile of the standard normal distribution. If the standard scale is shifted leftward about *d*′/2, the means of the noise distribution and the signal plus noise distribution shift toward −*d*′/2 and *d*′/2, respectively, and the fair response criterion is located at the origin. By calculating the response criterion *c* on this scale

(2) $$\begin{array}{l} c=-\frac{z\left(Hit\right)+z\left(FA\right)}{2} \end{array}$$

response bias can be evaluated, since positive values of *c* indicate that the observer prefers “different” responses, while negative values indicate that she/he prefers the “same” response category (see Appendix A in Supplementary Material).

An alternative bias measure can be defined in terms of the error proportion of wrong “different” responses:

(3) $$\begin{array}{l} q=\frac{Miss}{Miss+FA}. \end{array}$$

If *q* = 0.5, then both responses occur with equal likelihood. A ratio of *q* > 0.5 indicates a tendency to respond “different” while *q* < 0.5 indicates a preference toward “same” responses.

To compare response preferences for congruent and incongruent trials we also calculated odds ratios for both types of errors, i.e.,

(4) $$\begin{array}{l} OR=\frac{Miss/Hit}{FA/CR}. \end{array}$$

The OR indicates how much larger the odds are for wrong “different” responses compared to wrong “same” responses.

### 2.7. Statistical power analysis

For the context congruency paradigm there are currently no meta analysis data for the congruency effect. However, for the related composite effect, a recent meta analysis of 48 studies using the CD showed an average effect size of $\eta_{p}^{2}=0.32$ for the congruency × alignment interaction found in ANOVA (Richler and Gauthier, 2014). Expressed in terms of Cohen's effect size measure *d* the proportion of explained variance transforms to approximately *d* = 1.37. Here, the relations *d* = 2*f* and $f=\sqrt{\frac{\eta^{2}}{1-\eta^{2}}}$ were used (Cohen, 1988, p. 276 and p. 284). The CB, though, can be expected to have considerably smaller effect size. So far, there are currently no estimates available for bias measures. Since we could expect further attenuation of the CE and the CB due to inversion, we calculated a power analysis to estimate critical sample sizes for small (*d* = 0.2), medium (*d* = 0.5), and large (*d* = 0.8) effects, following Cohen's taxonomy (Cohen, 1988, p. 40). Since both the CE and the CB are defined as difference measures for both congruency conditions, the analysis was executed for a paired *t*-test, assuming same variance in congruent and incongruent conditions and a medium correlation of ρ = 0.5 among them. Further, the settings for the power goal were 1−β = 0.95 and α = 0.05. The results showed critical sample sizes of *N*_*c*_(*d* = 0.8) = 23, *N*_*c*_(*d* = 0.5) = 54, and *N*_*c*_(*d* = 0.2) = 327. Detailed analysis (see Appendix D in Supplementary Material) showed that the critical sample size for the given power goal accelerates remarkably when the effect size falls below *d* = 0.3. To be able to detect potentially weak CB effects for inverted presentation with a plausible power goal we decided to use a large sample of *N* = 319 subjects.

### 2.8. Data analysis

There were *n* = 16 replications of each trial type, “same” or “different”, respectively. If CR or Miss rates were zero or unity, they were corrected to 1/(2*n*) and 1 − 1/(2*n*), respectively, before *d*′ data were calculated (MacMillan and Creelman, 2005, p. 8). Both the *d*′ and the *c* data were analyzed with ANOVA, having Orientation (upright or inverted) and Congruency (congruent or incongruent) as repeated measures factors. Congruency effects were calculated from the *d*′ data by taking the difference *CE* = *d*′(*congruent*) − *d*′(*incongruent*), and the congruency bias was calculated according to *CB* = *c*(*incongruent*) − *c*(*congruent*). To control for outliers due to floor performance cases entered the statistical analysis if performance in the congruent condition was beyond 0.5 *d*′ units. This criterion excluded 16 of the 319 subjects. Thus, the final analysis sample comprised *N* = 303 cases. Data were analyzed using ANOVA with stimulus orientation (upright, inverted) and congruency (congruent, incongruent) as repeated measurements factors. For all analyses STATISTICA 13.0 software (Statsoft inc.) was used.

## 3. Results

**Figure 2:**
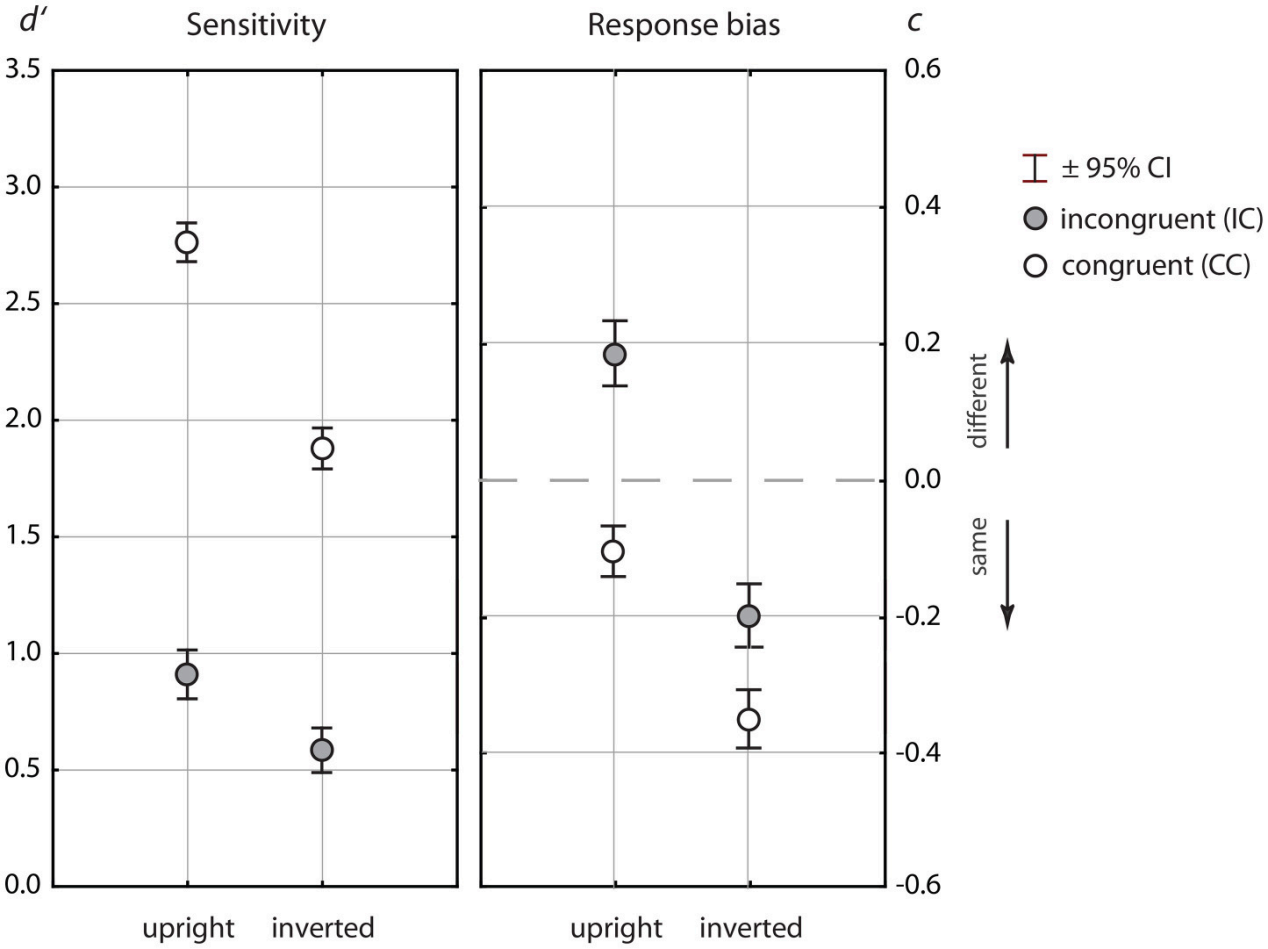
Mean sensitivity measure *d*′ **(left panel)** and response bias, measured by the estimated response criterion, *c* **(right panel)**. Data for upright presentation are shown left, and right for inverted presentation. Means for the congruent (open symbols) and the incongruent condition (filled gray symbols) are shown stacked to illustrate the effects of congruency.

Figure 2 shows the results for the sensitivity measure *d*′ and the response criterion *c*. The Tables 1, 2 show the ANOVA results for both measures. In both experimental paradigms there were large effects of congruency, both in the sensitivity measure and in the bias measure. Further, there were strong main effects of orientation, and strong orientation × congruency interactions in both measures.

**Table 1:** ANOVA results for testing the sensitivity measure, *d*′.

**Source of variation**	**SS**	**df**	${\hat{\sigma }}^{2}$	***F***	***p***	$\eta_{p}^{2}$
Orientation	104.6	1	104.6	309.21	<0.001	0.506
Error	102.2	302	0.3			
Congruency	737.5	1	737.5	1024.92	<0.001	0.772
Error	217.3	302	0.7			
Orientation × Congruency	22.8	1	22.8	78.66	<0.001	0.207
Error	87.4	302	0.3			

*The table shows source of variation, sum of squares (SS), degrees of freedom (df), variance estimate (${\hat{\sigma }}^{\text{2}}$), F- ratio, (F), significance level, p, and partial eta-squared, $\eta_{p}^{\text{2}}$*.

**Table 2:** ANOVA results for testing response bias with the response criterion, *c*.

**Source of variation**	**SS**	**df**	${\hat{\sigma }}^{2}$	***F***	***p***	$\eta_{p}^{2}$
Orientation	30.1	1	30.1	244.61	<0.001	0.448
Error	37.1	302	0.1			
Congruency	14.9	1	14.9	230.29	<0.001	0.433
Error	19.6	302	0.1			
Orientation × Congruency	1.4	1	1.4	20.98	<0.001	0.065
Error	20.2	302	0.1			

*The table shows source of variation, sum of squares (SS), degrees of freedom (df), variance estimate (${\hat{\sigma }}^{\text{2}}$), F- ratio, (F), significance level, p, and partial eta-squared, $\eta_{p}^{\text{2}}$*.

Exploring the sensitivity data showed average sensitivity of *d*′ = 2.74 for upright presentation in congruent contexts, which corresponded to a percent correct rate of *P*_*c*_ = 91.4% (see **Table 4**). Performance declined strongly for inverted presentation [*d*′ = 1.88, *P*_*c*_ = 81.2%] and the difference was highly significant [*F*_(1, 302)_ = 388.6, *p* < 0.001]. In incongruent contexts performance differences for upright and inverted presentation were not so pronounced [upright: *d*′ = 0.91, *P*_*c*_ = 67.3%; inverted: upright: *d*′ = 0.60, *P*_*c*_ = 61.5%], but also significant [*F*_(1, 302)_ = 43.9, *p* < 0.001]. Analysis of the response criterion *c* reflected a “different” bias in incongruent trials for upright presentation, and a “same” bias for congruent trials. In inverted presentation, there was a “same bias” in both congruency conditions^4^. As it was found for the sensitivity measure, there were inversion effects in the bias measure in congruent [*F*_(1, 302)_ = 200.9, *p* < 0.001] and incongruent contexts [*F*_(1, 302)_ = 116.8, *p* < 0.001] while the IE in congruent contexts was more pronounced.

The significant orientation × congruency interactions, observed in both measures, did not only indicate different inversion effects for both congruency relations, but also that both the CE and the CB were significantly larger for upright compared to inverted presentation. Table 3 lists the CEs and the CBs for both orientations. Albeit attenuated by inversion, the CE reached large effect size in Cohen's *d* measure for both orientations. The CB was considerably smaller, yielding a large effect size in upright and an effect of medium size in inverted presentation. Calculating the critical sample size necessary for a power of 0.95 at an effect size of *d* = 0.4 resulted in *N*_*c*_ = 84 subjects, which means that the given sample size of this study was large enough to substantiate a CB effect for inverted presentation (see Appendix C in Supplementary Material). To compare effect sizes across measures, we calculated confidence intervals for Cohen's *d*, using an estimate of its standard error (see Hedges and Olkin, 1985, p. 86):

(5) $$\begin{array}{l} \sigma_{d}=\sqrt{\frac{N_{0}+N_{1}}{N_{0}N_{1}}+\frac{d^{2}}{2\left(N_{0}+N_{1}\right)}}. \end{array}$$

**Table 3:** Congruency effects (CEs) and congruency bias effects (CBs).

**Measure**	**Orientation**	**Δ**	***s*_*e*_**	***t***	***p***	***d***	**σ_*d*_**	**CI*(d)***	***N***
Sensitivity (*d*′)	Upright	1.83	0.059	30.93	<0.001	1.78	0.096	[1.59,1.97]	303
Sensitivity (*d*′)	Inverted	1.29	0.056	22.93	<0.001	1.32	0.090	[1.14,1.49]	303
Response bias (*c*)	Upright	0.29	0.021	13.69	<0.001	0.79	0.084	[0.62,0.95]	303
Response bias (*c*)	Inverted	0.15	0.020	7.52	<0.001	0.43	0.082	[0.27,0.59]	303

*The table shows the difference measure, Δ, (the CE for sensitivity and the CB for response bias), its standard error, t- statistic for the paired test, significance level, Cohen's d, its standard error, its confidence interval, and number of observations*.

In Equation (5), *N*_0_ and *N*_1_ are the sample sizes, which are *N* = 303 each. The confidence interval for *d* is given by CI = *d*±*z*_(1−α/2)_σ_*d*_. For the CIs shown in Table 3 the alpha level was set to 5%. The results showed that the effect size of the CE was significantly larger in upright [*d* = 1.83] compared to inverted presentation [*d* = 1.29], since there was no overlap of the CIs for Cohens's *d*. The same result was obtained for the CB [upright: *d* = 0.79; inverted: *d* = 0.43, no overlap of CIs for *d*]. Across measure comparisons of effect sizes for the CE and the CB showed substantially larger effect sizes for the CE, and no overlap between the 95% confidence intervals (see Table 3).

We also tested whether there was overall bias in either orientation. For upright presentation there was a negligible overall “different” bias (*q* = 0.529), which proved to be significant [*c* = 0.039, *F*_(1, 303)_ = 4.32, *p* < 0.05]. For inverted presentation there was a large overall “same” bias (*q* = 0.336), which was also significant [*c* = −0.276, *F*_(1, 303)_ = 13.66, *p* < 0.001], indicating that the observers missed diagnostic cues for facial difference in upside down faces.

Table 4 shows the relative frequency data for both tests, which reflect the response preferences and their modulation by the congruency relation in detail. In both orientations the odds for wrong “different” responses were smaller than the odds for wrong “same” responses in congruent trials, and substantially larger in incongruent trials (see OR in last column of Table 4). In incongruent trials the rates of both error types increased (see columns FA and Miss), but the rates for wrong “different” responses (misses) increased much more. This stronger increase of erroneous “different” responses tripled the odds ratio for this error type in incongruent, compared to congruent trials for the upright orientation, and scarcely doubled its odds ratio for inverted presentation.

**Table 4:** Relative frequency data.

**Orientation**	**Congruency condition**	**CR**	**FA**	**Hit**	**Miss**	***P*_*c*_**	***q***	**OR**
Upright	Congruent	89.7	10.3	93.0	7.0	91.4	0.40	0.66
Upright	Incongruent	73.9	26.1	60.7	39.3	67.3	0.60	1.83
Inverted	Congruent	72.2	27.8	90.2	9.8	81.2	0.26	0.28
Inverted	Incongruent	54.0	46.0	69.0	31.0	61.5	0.40	0.53

*The table shows the rates for correct rejection, false alarm, hit and miss, as well as proportion correct rate, P_c_, as percent values. The last two columns shows the bias measure q, and the odds ratio, OR, for misses relative to false alarms*.

Albeit methodological reservations concerning the reliability of difference scores (Lord, 1963; Overall and Woodward, 1975) we calculated the bivariate correlation of the CE and the CB to prove whether association of both measures could be established on the level of individual subjects. For the upright orientation we obtained a modest but significant product-moment correlation [*r* = 0.24, *p* < 0.001], while a correlation was lacking for inverted presentation [*r* = 0.07, *p* = 0.224].

## 4. Discussion

In the Introduction it was outlined that congruency effects are expected in both sensitivity (CE) and bias (CB) if faces are processed holistically while observers attend to the internal facial features. Results obtained from a comparably large sample of young adult observers confirmed this prediction. We observed a large CE and a smaller, but substantial CB. Face inversion attenuated both the CE and the CB, but both remained pronounced effects. Hence, the obtained result patterns for upright and inverted presentation differed quantitatively, but not qualitatively. In the following we turn to the salient discrepancy in the effect sizes of the CE and the CB, touch recent quarrels of the design issue (Richler and Gauthier, 2013; Rossion, 2013), and discuss the context congruency and the composite face paradigm (Young et al., 1987) with respect to a potential transfer of our findings for measuring holistic integration among upper and lower inner face parts.

### 4.1. The CE and the CB capture different aspects of the failure of selective attention to parts

The CE reflects that more errors are made in incongruent compared to congruent trials, but it does not differentiate the kind of errors. Among all errors that are potentially more frequent incongruent trials, a large proportion may be due to holistic integration. The remainder may have other potential roots, such as failure of focal attention, fluctuations in the accuracy of face detail perception, or response conflict. This means that the CE may reflect mostly holistic, but also some non-holistic effects. As a result, it may overestimate the effects of holistic integration to certain degrees^5^.

The CB reflects that the observer makes more wrong “different” responses (i.e., more errors in “same” trials, misses) than wrong “same” responses (i.e., errors in “different” trials, false alarms) in incongruent, compared to congruent face context^6^. This is expected if the perceived properties of the wholes rather than of the parts drive the observer's responses, since all “wholes” are different in incongruent trials, while, in congruent trials, there is parity of same and different “wholes.” Integrating external and internal features into one holistic face representation results in erroneous perception of different personal identities (see the “same” trial example for the incongruent condition in Figure 1B). Exactly this has been exemplified in the “Presidential illusion” (Sinha and Poggio, 1996). The distinctiveness of the “illusion” of different identities varies somewhat with the distinctiveness of the external features (Andrews and Thompson, 2010), but, overall, we can expect a quite strong perceptual effect that induces a strong readiness of the observer to respond “different” in incongruent “same” trials. Errors in incongruent “different” trials (see Figure 1B) are not expected to increase to similar degrees, since the wholes and the attended parts are different. The attentive observer may notice that there is neither part-based, nor overall fit of the two faces—up to some cases where the internal features of the two face instances are highly similar. This, however, may be true only for a small portion of the face set^7^. Therefore, holistic integration of external and internal features predicts that the most frequent errors are misses in incongruent trials. This means that a CB is expected from holistic integration.

Table 4 shows that, in the experiment, errors of *both* kind, wrong “same” (false alarm) and wrong “different” (miss) responses, were more frequent in incongruent, compared to congruent trials. However, disproportionately more errors were wrong “different” responses. The disproportionate increase of misses in incongruent trials was somewhat attenuated for inverted presentation, however, qualitatively, the results patterns agreed for both orientations.

It is an advantage of the CB that it differentiates the two error types, reflecting the larger proportion of misses in incongruent compared to congruent trials, which is predicted by holistic integration of external and internal features. However, it is not possible to generalize to single trials. Certainly, not every miss is induced by holistic integration, some may occur for alternative reasons. The reverse is true for false alarms. In most of the cases, these errors will have alternative roots (see 2nd paragraph of this section), but for faces with highly similar internal features false alarms may arise due to holistic influence from same external features. For these reasons it is not possible to regard the CB as a “pure” marker of holistic processing. However, only the CB reflects whether a quite specific prediction from holistic processing holds, or not. As the comparison of effects sizes for CE and CB reveals (see Table 3), the CB is significantly smaller than the CE. Evaluating the CB along with the CE enables us to reveal whether wrong face identity perception, induced by different integrated wholes, is a significant effect in the data.

The CB effect as a portion of the CE could be reflected on the level of individual subject data by showing a correlation among measures. We just found a disappointing modest correlation in upright and practically uncorrelated measures in inverted presentation (section 3). However, a difference measure suffers from the problem of measurement error summation, which may preclude to find proof for association due to the unreliability of difference scores. The same problem was encountered in attempts to predict face recognition from the composite effect, defined as a difference measure or a regression residuum (Richler et al., 2014)^8^.

### 4.2. The CB is diagnostic of holistic processing only in the complete design

In a recent debate about the design issue in the composite face paradigm Rossion (2013) suggested to use an experimental arrangement following the partial design, and to analyse only “same” trials, since he considered the error in “different” trials as not diagnostic of holistic integration. As he argued, the effect of holistic integration could best be captured if composite faces are used in the aligned and the misaligned variety, comparing accuracy in “same” trials across the two alignment conditions, since only the alignment effect on “same” trials reflects the effects of holistic processing^9^.

It is true that a strong conflict of part-based and whole agreement is given only in incongruent “same” trials (see Figure 1, see section above). The problem with the suggestion to analyse only “same” trials is that there is a confound with a potential overall bias of the observer toward “different” responses. This confound is not resolved by introducing an additional non-aligned control condition, because aligned and non-aligned stimuli are physically distinct events on the display, and the observer may contingently adapt her/his decisional strategy to either condition. However, a sound experimental design must be able to segregate overall bias from congruency modulated bias.

A simple and straightforward solution for this problem is using the complete design. Measuring performance achieved in both “same” and “different” trials and from both response categories warrants bias-free measurement of performance. Second, the CD offers a way to revealing the effects of holistic stimulus processing Rossion and colleagues are aiming at, avoiding confounds with decisional strategy. While it may be true that the CE may overestimate the effects of holistic processing (see above)^10^, analysing the CB warrants to capture the increase in just the type of errors which are expected from holistic integration. Aligned composite stimuli in congruent and incongruent trials are not physically distinct events. The observer can therefore not adapt her/his decisional strategy contingently with the congruency relation. Consequently, significantly more “different” judgements in incongruent compared to congruent trials is diagnostic of responding to wholes rather than to parts. The CB reflects the increase of misses in incongruent trials, and, since it is a difference measure, it is not affected by the overall response bias of the observer.

### 4.3. Potential transfer to the composite face paradigm

At the time, the composite face paradigm and the context congruency paradigm have not yet been tested on the same set of stimuli, which confines any conclusions about potentially different results for CE and CB to across stimulus comparisons. Though, there are striking similarities of results when the CD was used. Richler et al. (2009) studied the effects of exposure duration on the composite face effect and response bias, using the CD. They found that both the CE and the CB developed until stable performance was reached in congruent and incongruent trials after about 200 ms presentation time. Most important, both effects then remained constant at strong levels across a large range of durations up to 800 ms, independent of whether the congruency relation was manipulated at test or at study. In another recent study the authors (Richler et al., 2011) studied the effects of inversion on CE and response bias. For larger presentation times (Experiment 1) results were highly similar to the results reported here. Authors observed both a large CE and a CB in upright presentation. Both effects were attenuated, but not abolished by inversion. Presenting face halves misaligned crucially diminished both the CE and the CB, and erased the CB for inverted presentation. Testing shorter presentation times (Experiment 2) proved a CE and a CB even for very brief timings (50 ms) in upright, while both measures critically depended on longer timings for inverted presentation. Hence, both our results and the results of the Richler et al. (2011) study provide evidence that inversion does not change the overall results pattern, but changes congruency effect and bias just quantitatively. The results therefore support the claim that face inversion does not change face processing qualitatively, with the same (holistic) mechanisms in play for both orientations, though with reduced efficiency when faces are inverted (Sekuler et al., 2004; Riesenhuber and Wolff, 2009; Richler et al., 2011). A quantitative account of the face inversion effect is further corroborated by recent findings about spatial frequency tuning of upright and inverted face identification (Willenbockel et al., 2010). In a reappraisal of former results indicating stronger reduction of the composite effect by inversion for lower compared to medium or higher spatial frequencies (Goffaux and Rossion, 2006, Experiments 2 and 3), the authors found proof for same dependency of face identification on spatial frequency for upright and inverted presentation^11^.

In the present study only a small set of face identities was used. This means that face parts occurred repeatedly, though always in different combinations. One might therefore surmise that subjects learn face parts by repeated presentation, and exploit this for matching just the inner parts, ignoring the facial surround. However, the results showed quite strong CE and CB effects, which means that the overall face context could not well be ignored, albeit there was a chance of learning diagnostic features. Further, separate results for the individual face identities agreed fairly well (see Appendix D in Supplementary Material). Results obtained for the role of feedback in the context congruency paradigm also show that, even with trial-by-trial feedback offering best prerequisites for perceptual learning (Herzog and Fahle, 1997), subjects were unable to ignore incongruent contexts, resulting in strong congruency effects, albeit the CE was attenuated in the feedback condition (Meinhardt-Injac et al., 2011, see there Figure 5). In a recent study Richler et al. (2015) systematically tested the effect of facial feature replication in the composite face paradigm, using just 5 faces to create composites, compared to using 95 faces without replication of face parts. The authors obtained practically identical results for the congruency effect (see Richler et al., 2015, Figure 3). These results indicate that holistic integration for faces is a robust effect which is hardly disrupted by feature knowledge.

Recent studies indicate that the CE and the CB vary with attentional conditions. Gao et al. (2011) used the CD to study the effect of priming local vs. global processing levels with Navon primes prior to composite face matching, and explicitly addressed the bias issue. They found a CE along with a CB in all priming conditions. Global priming increased the CE and the CB, while only the increase in the CE reached significance. Similarly, Meinhardt-Injac et al. (2017) found that using a global attentional focus in the composite face paradigm increased the CE and the CB for faces in children, adults and older adults, while for non-face control objects a CB was absent in all ages groups. A face-specific CB, however, was observed also for a narrow attentional focus, and in all age groups. Testing with the context congruency paradigm in four age groups from childhood to adulthood also revealed a face-specific CB at all ages (see Meinhardt-Injac et al., 2014, Figure 7). These results indicate a robust, face specific CB, albeit its magnitude may crucially depend on attentional conditions in the composite face paradigm^12^. Meanwhile, there are several studies using the CD for the composite face paradigm which report that holistic integration is modulated by attentional context conditions (Curby et al., 2013), or learned attention to parts (Chua et al., 2014, 2015).

The attentional cueing conditions used by Curby et al. (2013) encouraged grouping of upper and lower half, or not. Using the CD to compare faces to line draw stimuli with inherent Gestalt information Zhao et al. (2016) showed large CEs for the Gestalt-like non-face patterns, which were even larger than the CEs found for faces. For dot-patterns that were harder to group smaller CEs resulted. These results are important, because they indicate that inherent Gestalt information, besides expertise (Gauthier et al., 2003), can drive holistic processing. Authors communicated^13^ that also a large CB was observed for the Gestalt patterns, and a similar one for faces. The response criterion was located at about 0.3 standard units in incongruent trials and was about zero in congruent trials, which is a CB in the order of magnitude observed in this study. In the study of Zhao et al. (2016) it was necessary to construct at least two lower half mates for each upper half such that perceptual fusion of upper and lower halves was possible both in congruent and in incongruent trials. This points to the high relevance of having equal fusible lower halves in both trial types, a condition, which is usually not controlled when face halves are selected randomly (see footnote 10).

There are several potential reasons why the failure of selective attention may be different in the context congruency and the composite face paradigm. In the context congruency paradigm observers monitor internal features, while the external features change contingently with the congruency condition. In the composite face paradigm, the observers monitor a subset of the internal features (the upper or lower half), while the unattended subset changes contingently with the congruency relation. Results obtained with the context congruency paradigm suggest that observers focus the eyes/eyesbrows region, while the modulatory effect of context stems mostly from the external features. In Meinhardt-Injac et al. (2011) just the eyes/eyesbrows region was defined as the target region, while the remainder face regions formed the congruency modulated context. The results showed same CE and CB effects than for the whole set of internal features, having only external features as face context. These results indicate that differences in the lower parts of the internal features added little contextual modulation to the effect already exerted by the set of external features. This corresponds to recent neuroimaging results, which corroborate that the modulating effect of exchanging external features on facial representations in the FFA is strong (Andrews et al., 2010; Axelrod and Yovel, 2010, 2011).

Another difference is that swapped 3/4 views were used here, while usually frontal views are used in the composite paradigm. Changing views are effective for a deeper encoding of face identity (Duchaine and Nakayama, 2006), and have been shown to trigger cooperation and integration among neural populations of the Fusiform face area and the superior temporal sulcus (Lee et al., 2006), the former encoding face identity in a particular view (Grill-Spector et al., 1999; Andrews and Ewbank, 2004) and the latter encoding changes in face view but not in face identity (Andrews and Ewbank, 2004). Deeper neural processing for comparing faces across views is also indicated by modest age-related decline for sequential face matching within the same view, but considerable decline for changing views (Habak et al., 2008). While our 3/4 swapped views generated from the same original pictures did not convey different facial information, swapping of 3/4 views cancels face adaptation effects, and involves different view-selective neuron populations (Jeffery et al., 2006). In a recent meta analysis of 48 studies using the composite face paradigm the average CE was estimated to amount 1.37 units in Cohen's *d* (Richler and Gauthier, 2014, see section 2), while the CE in the congruency paradigm measured here was larger (see *d* and its CI estimates in Table 3). View change could be one important source for a stronger CE, since changing views enforces observers to stronger rely on face identity rather than on single face parts, which can more readily be grasped when the same view is repeatedly presented (Meinhardt-Injac et al., 2009).

Results obtained with the composite face paradigm show that the modulatory effect of the usually less salient lower face half (Davies et al., 1977; Haig, 1985; Barton et al., 2001; Sekuler et al., 2004) is substantial when there are no external features. However, in the composite face paradigm the viewing strategy of the observer is more complex and requires to follow predefined attentional constraints. If the upper face half is predefined as the target half, the observer can use an artificially narrow focus on faces by monitoring constantly the same image parts at study and test. To circumvent this, Richler and colleagues have devised a modification with upper and lower face half matching, whereby a cue presented after the study image informs the observers whether top or bottom halves are to be matched (Richler et al., 2008, 2009, 2011). This modification warrants that the whole face is encoded at study, while the observer tries part-based matching for the cued half at test. This “late cue” modification results in stronger holistic effects compared to having the target half predefined (Meinhardt et al., 2014), which indeed suggests artificial narrowing of the attentional focus with predefined target half. On the other hand, the late cue condition requires to adapt the attentional focus within a trial. It therefore requires developed capabilities in attentional control, which may be a problem when different age groups are studied (Greenwood and Parasuraman, 1999; Meinhardt-Injac et al., 2017). While it is true that it is hardly possible to obtain measures of holistic integration free of the influence of attentional constraints, the CE and the CB are substantial also for the variety with predefined target half (Gao et al., 2011; Zhao et al., 2016). The measures clearly separate faces from non-face objects in the absence of expertise (Gauthier et al., 2003; Meinhardt-Injac et al., 2017), albeit results are better contrasting when late cueing is used (Richler et al., 2011, Experiment 2).

### 4.4. Conclusions

Using the complete design and testing a large sample of *N* = 303 subjects has shown that there are two types of congruency effects in the context congruency paradigm. The CE captures the performance advantage in congruent, compared to incongruent trials, and is a large effect with an effect size of about 1.8 units in Cohen's *d* measure. This effect reflects that more errors are made in incongruent than in congruent trials, irrespective of the kind or errors. The second congruency effect, the CB, reflects the stronger preference for “different” responses in incongruent, compared to congruent trials. This effect is consistently smaller than the CE, reaching effect sizes of about *d* = 0.8. However, the CB indicates an increase in the specific error that should increase if attended and unattended face parts are integrated holistically, while the alternative error has no conceptual link to holistic processing. Therefore, the CB rather than the CE characterizes the face processing mode qualitatively. We recommend analysing both effects, the CE and the CB, to describe holistic processing in the framework of the complete design. First applications in the composite face paradigm showed that, by doing so, differential results patterns with a CE accompanied or not accompanied by a CB were obtained, thus discriminating face and non-face perception in different age groups (Meinhardt-Injac et al., 2017). Due to the high relevance of response bias for understanding congruency effects both qualitatively and quantitatively, it is mandatory to use a fully balanced design like the CD. This framework offers bias-free measurement of performance for all experimental varieties which aim at measuring the effects of context features on target features in composite objects.

## Ethics statement

The study was conducted in accordance with the Declaration of Helsinki. In detail, subjects participated voluntarily and gave written informed consent for their participation. In addition, participants were informed that they were free to stop the experiment at any time without negative consequences, and that their data would be removed from the panel. The data were analyzed anonymously. All procedures were approved by the local ethics board of Johannes Gutenberg University Mainz.

## Author contributions

All authors contributed equally to conception and design of the study. MP and BMI conducted the experiments and data preparation. GM contributed data analysis and interpretation. All authors were involved in writing, preparation of the manuscript, and its final approval. All authors agree to be accountable for all aspects of the work in ensuring that questions related to the accuracy or integrity of any part of the work are appropriately investigated and resolved.

### Conflict of interest statement

The authors declare that the research was conducted in the absence of any commercial or financial relationships that could be construed as a potential conflict of interest.

## Footnotes

^1^Exactly this was found for older adults when tested with composite faces and non-face objects (Meinhardt et al., 2016). Albeit older adults were strongly biased toward “same” responses, there was a face-specific CB.

^2^See Richler and Gauthier (2014) for a discussion of other potential bias related effects that are induced by the use of unbalanced designs.

^3^Studying the dependency of the CE and the CB on exposure duration Meinhardt-Injac et al. (2011) showed that both effects reached stable levels with saturating overall performance after about 300 ms exposure duration (see Meinhardt-Injac et al., 2011 Figures 3, 5, 6). Studying the effects of exposure duration in the composite face paradigm yielded highly similar findings (Richler et al., 2009), but saturating performance was reached about 100 ms earlier. Note that sequences of two frontal view face images were displayed in the study of Richler and colleagues, while here swapped 3/4 views were used. See section 4.3.

^4^Note that judging whether the expected value 0 for bias free decision lies within the confidence interval of the mean *c* value is the test of significance for response bias. The confidence intervals shown in Figure 2 indicate that there is significant response bias in all conditions.

^5^Generally, any matching task is easier with full agreement/disagreement of object features (congruent) as with just part based agreement/disagreement (incongruent). This is indicated by the fact that a marginal CE is observed also for non-face control objects (Richler et al., 2011), or in the misaligned control condition (Richler et al., 2008), which reflects effects of independent part interaction, but hardly holistic effects. These effects exist, but are not substantial. As indicated by the alignment × congruency interaction, which is consistently observed for faces, the CE reflects face specific processing to substantial degrees. However, the exact contributions of face-specific and face-unspecific processing cannot be disentangled in the CE measure. See Rossion (2013), but also Richler and Gauthier (2013) on this issue.

^6^A CB means that the estimated response criterion, *c*, is larger in incongruent than in congruent trials. With an increasing value of *c* the proportion of misses rises at the costs of false alarms (see Figure 1 in Appendix A in Supplementary Material).

^7^Note that the “Presidential illusion” shows that different external features induce perception of different personal identities in faces with same internal features. There are no examples in the literature showing that same external features induce perception of same personal identities in faces with different internal features.

^8^Note that both the predictor and the criterion are difference measures here, which means that four error variance components enter, which might not be independent in our case (Williams and Zimmerman, 1977). Since the correlation among measures is theoretically limited by the single measure reliabilities (Lord and Novick, 1968), we have no good outlook to revealing association of two difference measures with correlation methods.

^9^See Rossion and Boremanse (2008) for an experimental arrangement and data analysis following this rationale. Note that “same” and “different” trials were not balanced, but occurred with different a-priory probability (30% “different” and 70% “same” trials). See also Richler and Gauthier (2013) for criticism.

^10^Rossion (2013) criticizes using the CE as a measure of holistic integration, arguing that it lacks a neutral baseline and merges errors of different origins.

^11^Note that Cheung et al. (2008) could replicate the results of Goffaux and Rossion (2006) when only “same” responses were analyzed, but not when the CD was used.

^12^Note that in Gao et al. (2011) and Meinhardt-Injac et al. (2017) a modification of the CD was used. The lower face halves of incongruent-same trials were not necessarily the same as the lower face halves in congruent-different trials. This was owed to the fact that, in a given face set, proper combination of a lower half with different upper halves is not warranted, and may occasionally give odd results. Whether this variation of the paradigm is significant for the results remains to be tested.

^13^Personal email communication of Dr. Zhao.
