# Supplementary material for: On Response Bias in the Face Congruency Effect for Internal and External Features
Source: Front Hum Neurosci. 2017 Oct 17;11:494. doi: 10.3389/fnhum.2017.00494 (PMC5651001; doi:10.3389/fnhum.2017.00494)
Supplement: Supplementary file 1 [file DataSheet1.pdf]

APPENDIX A: THE BIAS MEASURE C

Let us define the four events resulting from a 2×2 stimulus-response matrix with “same” and “different” as the response alternatives (see here Fig. 1) in terms of conditional probabilities:

$$\begin{array}{lcl} CR & = & P(\text{“different”}|D) \\ FA & = & P(\text{“same”}|D) \\ Miss & = & P(\text{“different”}|S) \\ Hit & = & P(\text{“same”}|S) \end{array}$$

According to the basic assumptions of signal detection theory, these probabilities derive from normal probability density function (likelihood functions), $f(x|D)$ and $f(x|S)$, with equal variance σ^2. For the difference of the means of both distributions we have

$$\Delta\mu = \mu_S - \mu_D = k - \mu_D + \mu_S - k$$

where k is the decision criterion on the sensory continuum x, which is assumed to be constant throughout all measurements (see Fig. 1). Dividing by σ

$$\begin{aligned} d' &= \tfrac{\Delta\mu}{\sigma} = \tfrac{k-\mu_D}{\sigma} + \tfrac{\mu_S-k}{\sigma} \\ &= z_D - z_S \end{aligned} \tag{1}$$

Here, z_D is the standard quantile of the criterion k relative to $f(x|D)$, and z_S is the standard quantile of k relative to $f(x|S)$. Since $z(p) = \Phi^{-1}(p)$, with Φ^{-1} the inverse distribution function (quantile function) of the standard normal distribution, (1) is given by

$$d' = z(CR) - z(Miss) \tag{2}$$

Using the symmetry property $z(1-p) = -z(p)$, (3) is often written as

$$d' = z(Hit) - z(FA). \tag{3}$$

Now, verify that standardization of x with respect to $f(x|D)$ maps $\mu_D \mapsto 0$ and $\mu_S \mapsto d'$, i.e.

$$z(\mu_D) = \tfrac{\mu_D-\mu_D}{\sigma} = 0 \qquad z(\mu_S) = \tfrac{\mu_S-\mu_D}{\sigma} = d'.$$

The standardization $z = (x - \mu_D)/\sigma$ may be shifted to a new origin, chosen as half the standardized distance of means, d':

$$z' = z - \frac{d'}{2}.$$

This scale is chosen to express the response criterion k on a transformed standard axis:

$$c = z_D - \frac{d'}{2}. \tag{4}$$

Recall that $z_D = z(CR) = -z(FA)$. Then, by using (3), (4) reads

$$c = -\frac{z(Hit) + z(FA)}{2} \tag{5}$$

(see MacMillan & Creelman, 2005, p. 29). On this scale, positive values of c mean that the response criterion is closer to μ_S, and negative values mean that it is closer to μ_D. The means transform $z'(\mu_D) = -d'/2$, and $z'(\mu_S) = d'/2$, respectively (see Fig. 1)

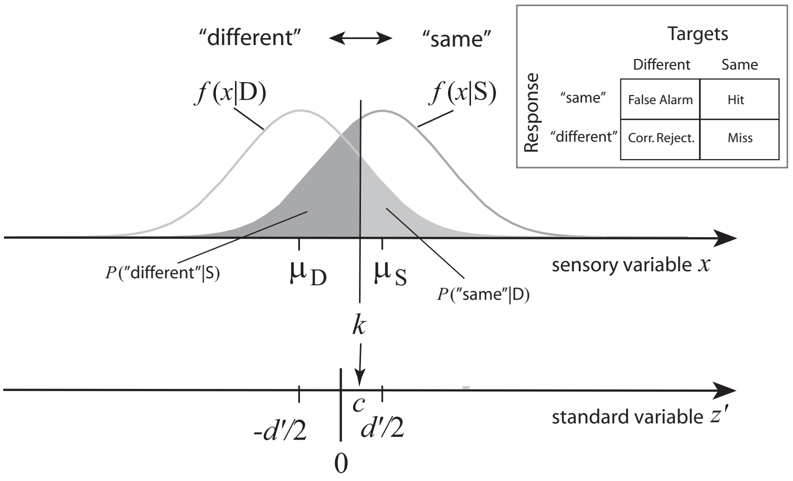

Figure 1. Likelihood functions $f(x|D)$, $f(x|S)$ as normal probability density functions with equal variance σ^2, decision criterion k, and corresponding probabilities of "false alarm" (P("same"$|D$)) and "miss" (P("different"$|S$)) events resulting from the position of the decision criterion k on the latent sensory continuum x. The lower continuum represents a transformed standard axis with $d'/2$ as the new origin. Expressed on this axis, positive values of the transformed decision criterion, c, correspond to more frequent "different" than "same" judgements, a *bias* towards the "different" response category (see arrow).

APPENDIX B: THE RESPONSE CRITERION C FOR MAXIMIZING PROPORTION CORRECT

Using a maximum a-posteriori probability decision rule for the states D and S, an observer responds "S" for a given value z of the standardized sensory continuum, if $P(S|z) > P(D|z)$. Since the a-posteriori probabilities $P(S|z)$ and $P(D|z)$ are given as Bayes probabilities, this decision rule reads

Choose "S" *if*

$$\frac{f(z|S)P(S)}{P(z)} > \frac{f(z|D)P(D)}{P(z)} \tag{6}$$

and "D" otherwise (see Green & Swets, 1966, chapter 1). After dividing both fractions,

Choose "S" *if*

$$LR_{SD}(z) = \frac{f(z|S)}{f(z|D)} > \frac{P(D)}{P(S)} \tag{7}$$

and "D" otherwise. Comparing (6) to (7) shows that the maximum Bayes probability decision rule is equivalent to a likelihood ratio decision rule, with $LR_{SD}(z)$ the likelihood ratio of the two likelihoods for z given S, and D, respectively. For convenience, one may set $\vartheta = P(D)/P(S)$ for the right hand side of (7). Assuming the likelihood functions to be Gaussians, the likelihood ratio becomes

$$LR_{SD}(z) = \frac{\exp\left(-\frac{z-\frac{d'}{2}}{2}\right)^2}{\exp\left(-\frac{z+\frac{d'}{2}}{2}\right)^2}. \tag{8}$$

Considering the likelihood ratio at the response criterion $z = c$ where $LR_{SD}(c) = \vartheta$, we take the logarithm in (8). This resolves to

$$LR_{SD}(c) = \ln(\vartheta) = cd' \tag{9}$$

Hence

$$c = \frac{\ln(\vartheta)}{d'}. \tag{10}$$

Now, if we set $P(S) = 0.25$, $P(D) = 0.75$ and assume $d' = 2.5$, inserting in (10) gives a value of $c = 0.44$. This means that the observer who uses a maximum a-posteriori probability decision rule would set the response criterion to a positive value on the standardized continuum of sensory states if D trials are more likely than S trials. This means that we should observe a "different" bias as a result of the attempt to compensate for a larger perceived likelihood of D trials compared to S trials.

APPENDIX C: SAMPLE SIZE CALCULATION FOR CE AND CB MEASURES

Because the CE and the CB are defined on individual differences obtained in congruent and incongruent conditions we performed a power calculation for a dependent sample (paired) t- test, using the power calculation module of STATISTICA 13.0. For calculating the critical sample size a power goal of 0.95 was chosen. The α level for testing the null hypothesis was set to $\alpha = 0.05$. Figure 2 shows the critical sample size as a function of effect size d.

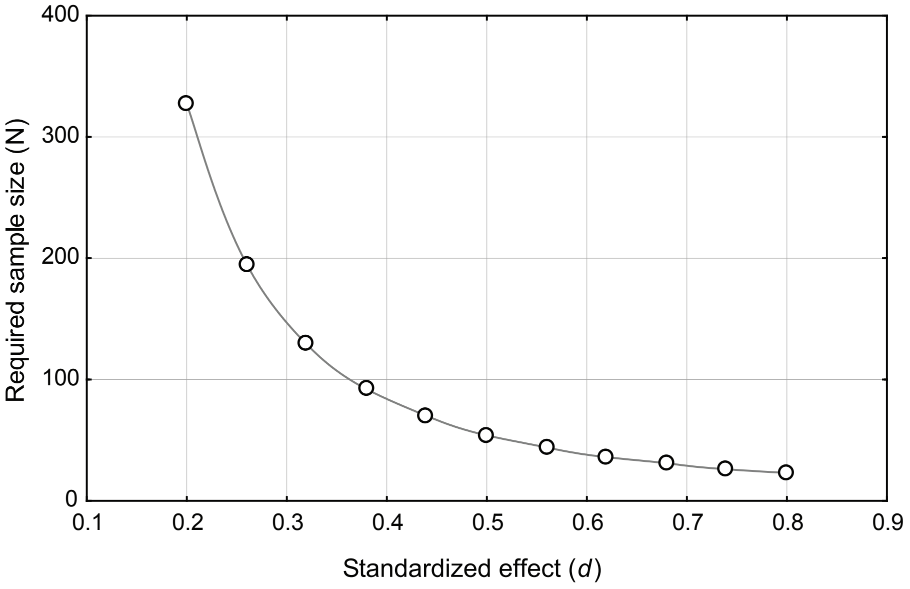

Figure 2. Required sample size as a function of Cohen's effect size index d for a paired t- test, assuming $\alpha = \beta = 0.05$.

APPENDIX D: RESULTS FOR SINGLE FACE IDENTITIES

Table 1 of this appendix shows separate results for the four face identities. Because the average number of replications for each identity in "same" and "different" trials, $\overline{n} = 4$, was too small to allow for a sound calculation of relative frequencies for each subject, the frequency data for Hit and CR were counted across

subjects, and the rates were calculated afterwards. Signal detection measures were computed from these rates.

Table 1. Results for the four face identities. The table shows hit, correct rejection and accuracy rates as percent values, the bias measure, q, sensitivity measure, d', estimated response criterion, c, and CE and CB congruency effect measures.

Orientation	Face identity	congruency	Hit	CR	P_c	q	d'	c	CE	CB
upright	F1	CC	94.4	89.9	92.2	35.8	2.86	-0.15	1.91	0.38
		IC	60.1	75.8	68.0	62.2	0.96	0.22		
	F2	CC	91.6	87.6	89.6	40.4	2.53	-0.11	1.63	0.22
		IC	63.4	71.1	67.2	55.9	0.90	0.11		
	F3	CC	91.8	85.9	88.8	36.8	2.47	-0.16	1.94	0.18
		IC	59.4	61.5	60.4	51.3	0.53	0.03		
	F4	CC	91.7	86.2	89.0	37.5	2.48	-0.15	1.95	0.40
		IC	50.5	69.4	60.0	61.8	0.52	0.25		
Mean									1.86	0.29
upright	F1	CC	88.4	76.1	82.3	32.8	1.90	-0.24	1.54	0.15
		IC	60.7	53.6	57.2	45.9	0.36	-0.09		
	F2	CC	85.2	70.5	77.8	33.3	1.58	-0.25	1.10	0.11
		IC	65.0	53.9	59.4	43.2	0.48	-0.14		
	F3	CC	90.8	75.7	83.3	27.5	2.03	-0.32	1.19	0.19
		IC	70.6	61.4	66.0	43.2	0.83	-0.13		
	F4	CC	88.3	73.6	80.9	30.8	1.82	-0.28	1.22	0.14
		IC	66.9	56.3	61.6	43.1	0.60	-0.14		
Mean									1.26	0.15
